# 5′ tRNA Halves: The Next Generation of Immune Signaling Molecules

**DOI:** 10.3389/fimmu.2015.00074

**Published:** 2015-02-19

**Authors:** Joseph Mohsen Dhahbi

**Affiliations:** ^1^Biochemistry Department, University of California Riverside, Riverside, CA, USA; ^2^Center for Genetics, Children’s Hospital Oakland Research Institute, Oakland, CA, USA

**Keywords:** 5′ tRNA halves, small non-coding RNAs, circulating 5′ tRNA halves, extracellular small non-coding RNAs, tRNA-derived small RNAs

Non-coding small RNAs including tRNA, rRNA, snoRNA, and Y RNA, have been recently shown to undergo processing into smaller RNA molecules (1, 2). These derivatives of known small RNAs are not merely degradation products but are specific cleavage products that function in patho-physiological conditions (3–5). Particularly, tRNAs are processed into two types of tRNA-derived small RNAs (2): (i) The 5′ and 3′ tRNA halves are 30–40 nt long and are produced by cleavage of mature cytoplasmic tRNAs (6). Two ribonucleases have been shown to cleave mature tRNAs near or in the anticodon loop to generate tRNA halves during stress: Rny1 in *Saccharomyces cerevisiae* (7) and angiogenin in higher eukaryotes (6, 8). (ii) The shorter tRNA-derived fragments (tRFs) are 18–22 nt long, and are produced from both mature and pre-tRNAs by Dicer or RNase Z. Here, I will consider only the tRNA halves and argue their potential as immune signaling molecules.

Initial reports showed that tRNA halves accumulate in *Tetrahymena thermophila* (9) and *Trypanosoma cruzi* (10) subjected to nutritional stress, and in *S. cerevisiae*, plants, and human cell lines where they become highly induced during oxidative stress conditions (8, 11). The stress-associated induction of tRNA halves has been suggested as a conserved feature of the cellular response to stress: 5′ tRNA halves are produced in the cytoplasm of stressed cells to inhibit translation and thus preserve cellular energy (8, 12). Many studies now indicate that some organisms and cell types express tRNA halves constitutively, while others produce them under stress conditions. For example, tRNA halves, in addition to other small RNAs derived from various RNA species, have been observed under non-stress conditions in plants (13), the soil bacterium *Streptomyces coelicolor* during its development (14), the fungi *Aspergillus fumigatus* in its resting state (15), and unstressed human cells (6, 16); however, these basal levels of tRNA halves are low and often increase during stress conditions (17). Also, it remains to distinguish them from intermediates of similar size generated during tRNA splicing (18). In other systems, tRNA halves were observed only under stress conditions [reviewed in Ref. (19)].

Fu and colleagues were the first to observe tRNA halves in mammalian tissues (6). In an effort to identify liver-specific miRNA, they found traces of Val–tRNA–AAC halves in fresh normal mouse liver and heart tissues, and much higher levels when these tissues were subjected to *ex vivo* starvation by incubation in PBS for various lengths of time (6). The same study also reported the presence of significant amounts of tRNA halves in human fetal liver tissue that has been kept at room temperature for a few hours before RNA extraction (6). Two other studies later reported the presence of mainly 5′ tRNA halves in mouse mature sperm (20) and in human semen (21). Our own examination of several mouse tissues revealed that 5′ tRNA halves are exceedingly more expressed in hematopoietic and lymphoid tissues than other tissues (22). We have found that mouse spleen, lymph nodes, and fetal liver, which is a hematopoietic tissue, leukocytes, bone marrow, and thymus, and human leukocytes contain considerable amounts of 5′ tRNA halves when compared to mouse non-hematopoietic tissues (testes, liver, heart, brain, and kidney), which showed only traces of 5′ tRNA halves [Figure 1; (22)]. The very small amounts of 5′ tRNA halves we detected in non-hematopoietic tissues are comparable to the levels observed by Fu and colleagues in normal fresh mouse liver and heart tissues [see Figure 2 in Ref. (6)]. We have speculated that the trace amounts of 5′ tRNA halves in non-hematopoietic tissues may originate from residual blood cells in those tissues (22). A later deep sequencing survey of several mouse tissues under physiological conditions found very low levels of 5′ tRNA halves in all tissues examined except the bone marrow, which expressed significant quantities of 5′ tRNA halves (23).

**Figure 1:**
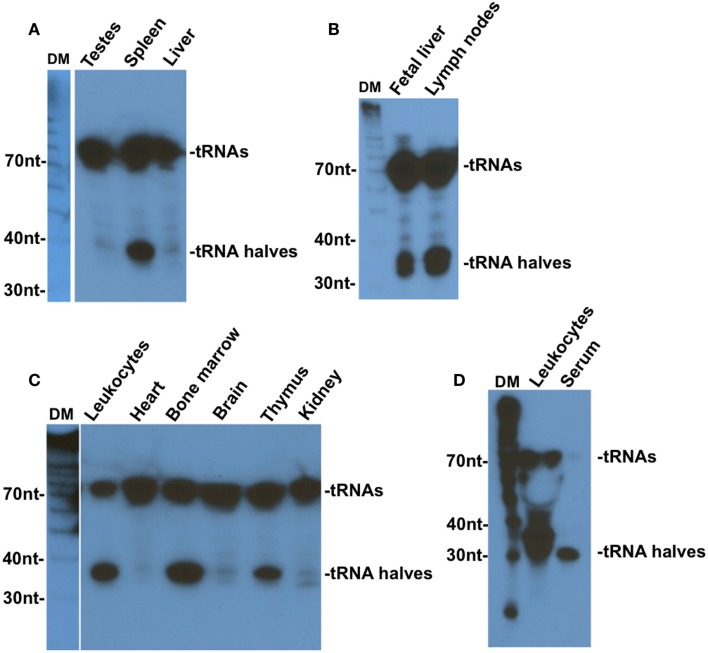
**Tissue distribution of tRNA–Gly–GCC halves**. Northern blotting analysis of RNA extracted from the indicated mouse tissues **(A–C)** or from human leukocytes or serum **(D)**. Blots were analyzed with 5’ end probes of tRNA–Gly–GCC. DM, decade markers. This figure is an edited version of Figure 7 in Ref. (22).

Small non-coding RNAs and their derivatives are released into the extracellular environment and thereby may carry paracrine and even endocrine signaling functions (22, 24–26). There is evidence that extracellular miRNAs can enter cells and alter gene expression and functions of the recipient cells (27), which suggests involvement of miRNAs in cell-to-cell communication not only in normal biology but also in disease pathogenesis (28). MiRNAs complexed to high-density lipoprotein entered hepatocytes and altered expression of genes involved in lipid metabolism, inflammation, and atherosclerosis (27). Extracellular miR-126 secreted by endothelial cells triggered the production of the chemokine CXCL12 in recipient vascular cells (29). Similarly, miR-150 secreted by human blood cells and cultured monocytic THP-1 cells reduced c-Myb expression and enhanced cell migration after delivery into HMEC-1 cells (30).

Using deep sequencing of serum small RNAs, we and others detected 5′ tRNA halves circulating in mouse and human bloodstream (31, 32), they were later found in rat and monkey serum at levels higher than miRNAs (23). Likewise, extracellular tRNA halves were observed in another biological fluid, human semen (21), media surrounding cell lines (33), and in plant phloem sap (34). Extracellular tRNA halves have no known functions yet; however, it is important to have an open mind regarding the function of circulating 5′ tRNA halves. Their intracellular counterparts have been suggested to act as signal molecules in stress-induced response (4, 35). Specifically, cellular 5′ tRNA halves promote assembly of stress granules and inhibit translation in mammalian cells (8, 12, 36). Stress-induced 5′ tRNA halves act independently of the eIF2α phosphorylation pathway; instead, they inhibit translation by associating with the translational repressor YB-1 and displacing eIF4G/eIF4A from the translation initiation complex (12). A recent study has discovered an additional signaling function of tRNA halves during osmotic stress: they protect cells from apoptosis by sequestering cytochrome *c* and thus inhibiting apoptosis (37). Furthermore, it has been shown that blocking the formation of tRNA-derived small RNAs by inhibiting tRNA cleavage slows tumor development (38). The involvement of intracellular tRNA halves in such key biological processes (5, 12, 37, 39) strongly suggests that they may be released in the circulation in a form that also has biological and functional significance. Given the evidence discussed above that extracellular miRNAs may enter recipient cells and modulate their functions, it seems likely that circulating 5′ tRNA halves could also act as cell-to-cell communication signaling molecules that enter recipient cells and alter their functional properties analogously to extracellular miRNAs. In support of possible functionality of circulating 5′ tRNA halves, we have found that aging alters the serum levels of specific subtypes of 5′ tRNA halves in mice while calorie restriction mitigates the age-associated changes (22); aging and calorie restriction were used as model physiologic changes to explore the functional potential of circulating 5′ tRNA halves. We also found that changes in serum levels of specific types of 5′ tRNA halves are associated with breast cancer and its clinicopathological characteristics (31). Levels of 5′ tRNA halves rapidly increased in the serum of mouse and monkey models of LPS-induced acute inflammation and in patients with active hepatitis B virus infection (23), and in the livers of humans and chimpanzees with chronic viral hepatitis (40). Furthermore, the production of tRNA fragments has been observed in several human pathological conditions including cancer, infection, and neurodegeneration [Reviewed in Ref. (39)].

Here, I propose that 5′ tRNA halves are potential systemic immune signaling molecules. I base this idea on the findings discussed above and summarized below:

(1) Intracellular 5′ tRNA halves are emerging as signaling entities.
(2) 5′ tRNA halves occur at the whole organism level and not only in cell lines.
(3) 5′ tRNA halves are drastically more expressed in hematopoietic and lymphoid organs relatively to other tissues, and concurrently circulate in the bloodstream as stable complexes.
(4) The expression of 5′ tRNA halves at the organismal level, mainly in immune tissues and in the bloodstream, takes place under normal, non-stressed physiologic states. This is in contrast to observations in cell lines where tRNA halves are generally produced in response to stress. Furthermore, both 5′ and 3′ tRNA halves are induced by stress in cell lines; while in whole mouse organism under normal physiologic conditions, predominantly 5′ tRNA halves are present in immune tissues and in serum.
(5) Finally, the circulating levels of 5′ tRNA halves can be modulated by patho-physiologic conditions.

Taken together, these findings highlight the relevance of 5′ tRNA halves in immunity and may be even in hematopoiesis, and hint that these tRNA-derived small RNAs could be secretory signals in a cell-to-cell communication system analogously to circulating miRNAs. There is some evidence in the literature that supports the idea that 5′ tRNA halves may play a role in immunity. Deep sequencing revealed that small RNAs derived from the 5′ and 3′ ends of mature tRNAs were abundant in the cytoplasm of immune cells and small RNAs derived mostly from the 5′ ends were selectively enriched in vesicles derived from these immune cells (33). 5′ tRNA halves were found in human seminal exosomes (21); seminal plasma and exosomes exert immunosuppressive effects on cells in the genital mucosa to induce tolerance to paternal antigens (41). Exosomes mediate inter-cellular communication by transferring its cargo, which includes small RNA molecules (42). Thus, the immunosuppressive effects of seminal exosomes may be at least in part mediated by the activities of 5′ tRNA halves.

When used as adjuvants of hepatitis B surface antigen in mice, tRNA fragments-induced Th1 and CTL responses through recognition of TLR3 (43). Bacterial tRNAs bind TLR7 through recognition of specific nucleoside modification patterns and induce secretion of IFN-α from immune cells (44). This mediation of humoral- and cell-mediated immune responses through stimulation of TLRs by small RNAs has been recently described for the most studied small non-coding RNAs, miRNAs (45). Tumor-secreted miR-21 and miR-29a bind to murine TLR7 and human TLR8 in immune cells and trigger an inflammatory response (45).

Specific nucleoside motifs within tRNA may act as structural anti-determinants for innate immune recognition; the stem-loop of human tRNA–Ala and an interaction between D and T loops of tRNA–His can be epitopes for autoantibodies found in serum of patients with idiopathic inflammatory myopathies (46). Treatment with fungal tRNA protected cells against adenovirus infection by inducing IFN-β synthesis (47). Incubation of cells with methionine initiator tRNA or the crude extract of plant tRNA induce IFN-α production (48). Infection with respiratory syncytial virus (RSV) induces 5′ tRNA halves in human airway epithelial cells by cleavage at the tRNA anticodon loop by angiogenin (5). This study further showed that 5′ tRNA–Glu–CTC half promotes viral replication while induction of chemokines and cytokines by RSV was significantly decreased upon inhibition of 5′ tRNA–Glu–CTC half by its anti-sense sequence. *T. cruzi* secretes tRNA halves in extracellular vesicles that can be delivered to host mammalian cells where the parasite tRNA-derived small RNAs induce regulation of genes involved in cell defense and immune responses against pathogens (49). This is a clear indication that tRNA-derived small RNAs could be relevant players in the host–pathogen signaling. Collectively, these observations point to a tRNA-derivatives characteristic that is conserved across various kingdoms of life.

Finally, another interesting observation is that the largest human tRNA gene cluster is located in the major histocompatibility complex (MHC), the genomic region that is crucial in adaptive and innate immunity. It has been suggested that clustering of tRNA genes in the MHC may allude to a tRNA role in the immune system (50). Other genes with immune-related functions, including inflammation and stress response genes, also co-localize with MHC (50). These observations provide further support the suggestion that 5′ tRNA halves may act as immune signaling molecules.

In summary, 5′ tRNA halves stably circulate in the bloodstream similarly to miRNAs, and given the increasingly recognized functions of extracellular miRNAs, it is not farfetched to envisage equally significant functions for the circulating 5′ tRNA halves. More intriguingly, unlike circulating miRNAs, which are secreted by all types of peripheral tissues, circulating 5′ tRNA halves seem to be concentrated in hematopoietic and lymphoid tissues, which strongly implies a role of 5′ tRNA halves in the immune system. This predominance of 5′ tRNA halves in hematopoietic tissues may further suggest a function in hematopoiesis, e.g., involvement in important stages such as the commitment and differentiation of stem and progenitor cells. I believe that this idea is worthy of investigation, given the great potential of extracellular RNAs as non-invasive biomarkers of health and disease. It is clear that much work is needed to test this idea. In particular, it remains to demonstrate that 5′ tRNA halves exert immune-related functions upon uptake by recipient cells in peripheral tissues. Future studies addressing the production, secretion, uptake, and functions of cellular and circulating 5′ tRNA halves will provide insights into the proposed role of 5′ tRNA halves in the immune and hematopoietic systems or other as yet undetermined functions. Establishing 5′ tRNA halves as new players in the complex processes of hematopoiesis and immunity would be invaluable for understanding hematopoietic disorders such as blood cancers and diseases related to immunity such as inflammatory disorders. Unraveling the mechanisms underlying the functions of 5′ tRNA halves will provide opportunities for discovering health and disease biomarkers and designing new therapeutic strategies.

## Conflict of Interest Statement

The author declares that the research was conducted in the absence of any commercial or financial relationships that could be construed as a potential conflict of interest.
